# Women’s economic empowerment and maternal mental health: A qualitative study in Rural Kenya

**DOI:** 10.1192/j.eurpsy.2024.1689

**Published:** 2024-08-27

**Authors:** C. W. Wainaina, E. Igonya, F. M. Wekesah, E. M. Sidze

**Affiliations:** African Population and Health Research Center, Nairobi, Kenya

## Abstract

**Introduction:**

**Background:** Maternal mental health is increasingly becoming a public health concern in developing countries because of predominant health and socio-economic inequalities. Mental well-being is essential for a woman to cope with daily life stresses and contribute positively to her community. Initiatives that empower women can enhance their well-being and improve the health of their families. However, limited evidence shows how women’s empowerment affects maternal well-being in a rural setting.

**Objectives:**

This paper explores the perspective of women’s economic empowerment in a rural Kenyan community and its effect on women’s mental well-being.

**Methods:**

We purposively sampled women and men from the rural community who met the eligibility criteria (women who were pregnant and or with a child less than two years old and married men and residents in the community. We conducted two focus group discussions with the men and women separately, 11 key informant interviews with community stakeholders, and a four-month participant observation of 20 women participants who were pregnant and or with a child less than one year old.

**Results:**

The study found that economically empowered women had greater decision-making power and self-efficacy. However, cultural expectations and barriers that dictated the role of women prevented them from accessing and controlling resources and participating in important decisions such as land and property ownership. Women faced domestic violence (physical, verbal, and denial of basic needs) and inadequate support (emotional, physical, and financial) from spouses and other family members. These challenges and barriers increased their mental stress. To cope, women engaged in economic activities individually or in groups to meet the basic needs of their families.

**Conclusions:**

Women’s economic empowerment can positively and negatively affect their overall well-being. Positively, women gain greater access to resources, improved decision-making, and the ability to plan and achieve their goals. Negatively, empowerment can lead to reduced spousal and kin support and an increased risk of domestic violence. Furthermore, these negative consequences can also affect women’s mental well-being. To ensure the well-being of mothers, it is crucial to engage men in empowerment programs and raise awareness in communities to address socio-cultural norms that impede women’s economic empowerment and negatively affect the well-being of women. Additionally, mental health support should be incorporated into these empowerment programs to mitigate the negative effects of women’s empowerment and improve resilience.

**Disclosure of Interest:**

None Declared

